# A Pilot Intervention Using Gamification to Enhance Student Participation in Classroom Activity Breaks

**DOI:** 10.3390/ijerph16214082

**Published:** 2019-10-24

**Authors:** Lexie R. Beemer, Tiwaloluwa A. Ajibewa, Gabriel DellaVecchia, Rebecca E. Hasson

**Affiliations:** 1 School of Kinesiology, University of Michigan, 1402 Washington Heights, Ann Arbor, MI 48109, USA; abeemer@umich.edu (L.R.B.); tajib@umich.edu (T.A.A.); 2 Childhood Disparities Research Laboratory, University of Michigan, 1415 Washington Heights, Ann Arbor, MI 48109, USA; 3 School of Education, University of Michigan, 610 E. University Avenue, Ann Arbor, MI 48109, USA; dellaveg@umich.edu; 4 School of Public Health, University of Michigan, 1415 Washington Heights, Ann Arbor, MI 48109, USA

**Keywords:** game design, classroom-based physical activity, intervention, motivation, children, school health

## Abstract

The purpose of this study was to determine the effect of adding game design elements (goal setting, feedback, and external rewards) to an evidence-based physical activity intervention to increase in-class physical activity participation (intensity of activity breaks performed). Nine third- through sixth-grade classrooms (*n* = 292 students) in one elementary-middle school in Detroit, Michigan (49% female, 95% nonwhite; 80% qualified for free/reduced lunch) participated in this 20-week intervention where teachers implemented 5 × 4 min moderate-to-vigorous activity breaks per day. Gamification of activity breaks occurred during weeks 13–20 of the intervention and included the use of game design elements and classroom goals for activity break intensity. Moderate-to-vigorous physical activity (MVPA) during activity breaks was measured via direct observation. There was a significant effect of intervention with a 27% increase in student MVPA participation during the gamified intervention weeks compared with the standard intervention weeks (*p* = 0.03). Gamification of activity breaks resulted in 55% (compared with 25% during the standard intervention) of students accumulating approximately 20 min of health-enhancing physical activity per day in their classroom. These findings provide preliminary evidence that gamifying activity breaks may be an important strategy for increasing student participation in classroom activity breaks.

## 1. Introduction

Despite the numerous health benefits associated with physical activity (PA), many children do not meet the national PA guidelines. Targeting classrooms, where children spend the majority of their school day in seated instruction, has provided a unique opportunity to increase youth PA and help schools achieve their local wellness policies. Classroom-based PA interventions are built around teacher-implemented academic lessons that are designed to incorporate 10–15 min of continuous PA as a complement to daily lessons [1,2,3,4]. Several programs have been developed that combine moderate-to-vigorous physical activity (MVPA) with teaching and academic content, while others have used MVPA as a short break from seated academic instruction to re-energize students [1,2,3,4,5,6,7,8,9]. While these interventions have demonstrated success in increasing in-school PA, as well as improving fitness, weight status, on-task behavior, and academic achievement in children, lower participation has been observed in students attending under-resourced schools [9]. For example, in the Interrupting Prolonged sitting with ACTivity (InPACT) intervention, student engagement in MVPA during an activity break was 77% in the under-resourced school compared with 88% in adequately resourced schools [9]. An important conclusion made by the authors was that additional strategies are needed to motivate behavior change in children attending under-resourced schools towards increased activity levels.

Goal-setting theory has been used to explain how to improve performance by setting and monitoring goals. In this context, a goal is defined simply as “what an individual is consciously trying to do” [10]. This theory has traditionally been used to improve performance in work-related tasks but has also been applied to motivate behavior change related to PA. For example, Horne et al. evaluated a goal-based reward and pedometer-feedback intervention on PA participation in children aged 9–11 years [11]. Children were rewarded with small incentives (e.g., balls and frisbees) for reaching their daily step count goal. This in turn motivated both girls and boys to take significantly more steps during the intervention compared with baseline step counts and the control group. Another aspect of goal-setting theory is that when given specific goals, individuals will be motivated to perform better than when given nonspecific or no goals [12,13]. Gao and Podlog tested the effects of three goal-setting conditions (easy, difficult, and best effort) on PA levels in first- through sixth-grade Latino students in an afterschool interactive dance program [14]. Students who set specific (easy or difficult) goals increased their PA levels more than the “do-your-best” group. Further, the group that set difficult goals had the highest improvement in PA levels. Taken together, these studies suggest that interventions that include specific goal-setting theory elements (i.e., goal setting, feedback, and external rewards) can have a positive impact on children’s PA levels.

An interactive way to establish goals and provide rewards to enhance children’s motivation and engagement in PA is through gamification. Gamification is the application of game design elements and principles applied in nongame contexts to motivate behavior change [15]. Similar to many activities that involve game play, gamification is inherently a goal-oriented activity. A real-world example of gamification used to increase PA participation is the interactive phone application Pokémon Go™. Pokémon Go™ encourages walking by prompting users to travel through an augmented reality collecting Pokémon characters for points and rewards. Althoff et al. assessed changes in PA participation among Pokémon Go™ users and after 30 days of playing the game; daily step counts increased 26%, equating to 1473 additional steps [16]. In the control group (individuals who did not use the application), daily step count decreased by 50 steps. Another example of gamification is the Behavioral Economics Framingham Incentive Trial (BEFIT), a 12-week randomized clinical trial where families in the gamification arm could earn points and progress through levels based on achieving personal PA goals [17]. Compared with the control arm, participants in the gamification arm had a significantly greater pre- and post-intervention increase in mean daily steps (1661 vs. 636 steps) [17]. 

Gamification is also a commonly used practice by school teachers to improve motivation, engagement, and student learning outcomes [18]. For example, Filsecker et al. used digital tokens as a reward to acknowledge individual accomplishments, including completing a project, mastering a skill, or exhibiting positive behavior in a group of fifth-grade students [19]. The authors observed that the use of external rewards did not undermine student motivation and students showed larger gains in understanding science [19]. Further, a study by Su et al. tested how gamification influenced learning, achievement, and motivation in three fourth-grade classrooms [20]. They demonstrated that students in the experimental group who used a mobile gamified learning system application had higher learning achievement than the two control groups that either used a smartphone to access materials or received traditional teaching without technology. Gamification has demonstrated success in the classroom to improve educational outcomes and may be a useful strategy to promote PA in this environment. However, interventionists have not explicitly used game design elements grounded in goal-setting theory to promote PA in the classroom. 

Given the lower participation in classroom activity breaks among children attending under-resourced schools, the purpose of this study was to determine the effect of adding game design elements (goal setting, feedback, and external rewards) to an evidence-based PA intervention, InPACT, to increase in-class PA participation (intensity of activity breaks performed). It was hypothesized that gamification of InPACT would increase student participation at the desired intensity level during the gamified intervention period compared with the standard intervention period. As an exploratory aim, we examined potential grade-level and gender differences in the effect of gamification on PA participation.

## 2. Participants and Methods 

### 2.1. Participants and Recruitment

The participating elementary-middle school was located in Detroit, Michigan. Nine third- through sixth-grade classrooms participated in this intervention, (two third-grade, two fourth-grade, two fifth-grade, and three sixth-grade). This school was selected based on their participation in Project Healthy Schools, a health promotion program housed in middle schools throughout the state of Michigan [21]. This school also met our definition of “under-resourced”, which was defined as ≥40% of students on free/reduced price lunch, which is consistent with the United States Department of Education eligibility criteria to qualify for Title I funding [22]. Before the start of the intervention, research staff met with the school principal to provide a detailed description of the study and to identify third- through sixth-grade teachers who may be interested in participating. Child participants who were in the third- through sixth-grade and able to participate in physical education class (which demonstrates ability to participate in various movements and physical activities) were eligible for participation in this project. 

This study was considered Exempt Human Research by the Institutional Review Board at the University of Michigan (HUM00137925) due to the following reason: “research conducted in established or commonly accepted educational settings, involving normal educational practice, such as (i) research on regular and special education instructional strategies, or (ii) research on the effectiveness of or the comparison among instructional techniques, curricula, or classroom management methods”. Therefore, consent was not required from parents and/or guardians. However, a letter both in the Spanish and English languages was sent home to parents describing the program and giving them an opportunity to opt out. Children were also given the opportunity to opt out of activity breaks and were instructed to sit quietly while the rest of the class participated in activity breaks. All students participated in the intervention, except for one student who opted out for religious reasons. A total of 292 students, aged 8–13 years (51% males; 49% females) participated in this intervention. Approximately 95% of students were nonwhite and 80% of the students were eligible for free or reduced-price lunch throughout the whole school [23,24]. 

The following PA opportunities were available at this school as reported by the physical education teacher: (1) one 50 min physical education class per week, led by a certified instructor (third- through fifth-grade); (2) recess for 20 min, 5 days per week (third- through sixth-grade); and (3) afterschool volleyball, soccer (sixth-grade), and track (third- through sixth-grade) sport teams. 

### 2.2. Study Design

InPACT was a 20-week classroom-based PA intervention. Teachers were asked to implement 5 × 4 min MVPA breaks per day, 5 days per week. Prior to the start of the intervention (September–November 2017), research staff conducted 3 × 1 h in-service teacher trainings (once per month) and provided implementation resources (i.e., teacher training manual, compendium of PA breaks, and classroom management posters) to enhance fidelity. Teacher check-in meetings were conducted once per month (January–May 2018) to answer questions, communicate updates, and troubleshoot problems that arose. 

To provide ease of implementation to teachers, the InPACT research team developed a compendium of PA in compliance with health-enhancing PA breaks for teachers to use and adapt for their classrooms. “The Compendium of Physical Activity and Youth” and “The Kinesthetic Classroom” were used as references for developing the InPACT compendium [25,26]. Activity break intensity was determined based on the American College of Sports Medicine’s guidelines and the Centers for Disease Control and Prevention (CDC) recommendations to classify MVPA as 60%–85% of heart rate maximum [27]. Because exercise intensity was a new concept to many teachers and students, InPACT exercise videos were also developed to help guide intensity using tempo and visual cues. The videos included research staff modeling the activities at the correct intensity, encouraging prompts to motivate students to continue exercising the entire duration, music, and a heart rate guide. A total of seven videos were developed for the intervention and were made accessible to teachers on the study website. Teachers were not required to use the Compendium or InPACT activity videos but were simply asked to ensure their students performed the prescribed number of activity breaks at a moderate-to-vigorous intensity. Type of exercises did not differ depending on the phase of implementation (ramp-up vs. standard vs. gamified). 

### 2.3. Intervention Timeline

InPACT implementation consisted of three phases: a ramp-up, a standard intervention, and a gamified intervention. *Ramp-up:* During weeks 1–4, teachers followed an incremental protocol where they were asked to complete a 1 × 4 min activity break per day during the first week and then to add a 1 × 4 min activity break each successive week until reaching 5 × 4 min activity breaks per day. The goal was to allow adequate time for teacher and student familiarity with InPACT and to establish classroom procedures. *Standard intervention:* During weeks 5–11, teachers implemented standard 5 × 4 min activity breaks per day without gamification components. PA participation was directly observed by research staff during this period and student participation rates during week 10 were used to establish baseline goals during the first week of the gamified intervention. *Gamified intervention:* During week 11, the InPACT research team visited each classroom to introduce the gamified protocol to students using an interactive video (https://youtu.be/dVkCJ07b0yA). Activity breaks observed during this introductory week were included as a gamified week in the analysis due to the video’s potential influence on student participation. Following spring break (week 12), during weeks 13–20, the competition began, and teachers implemented 5 × 4 min activity breaks per day using the gamified protocol. 

### 2.4. Gamification Protocol

**Figure 1:**
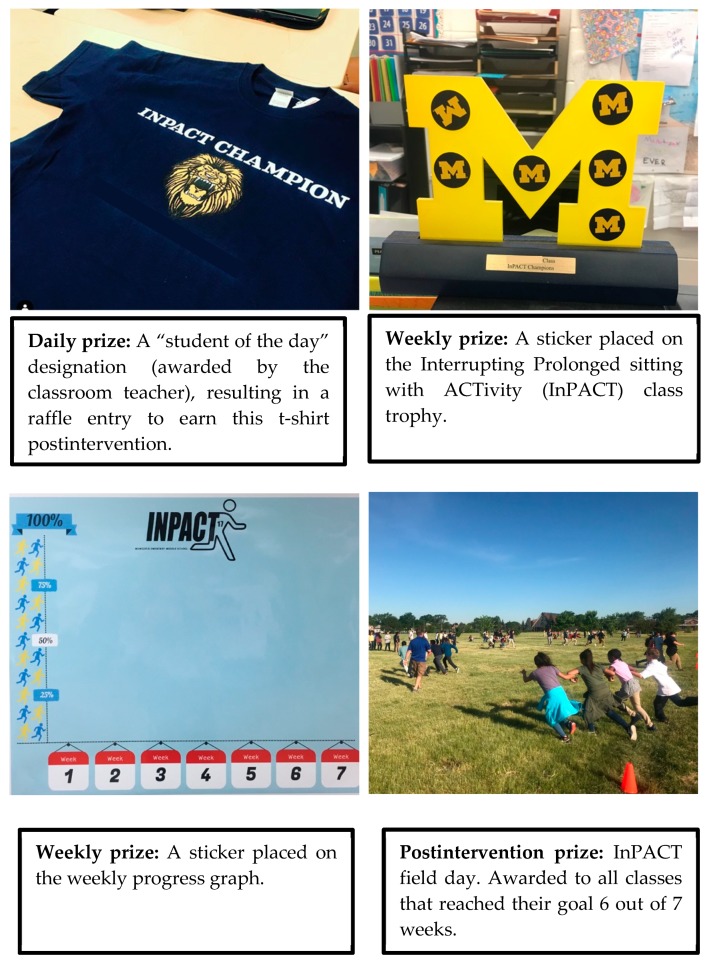
The gamified intervention daily, weekly, and postintervention prizes.

During gamified weeks, each classroom received challenging yet attainable weekly goals for students exercising in MVPA during an activity break (i.e., a 5%–10% increase in participation the following week, equating to one to two additional students engaging in MVPA). Goals were classroom specific and based on the average percentage of students exercising in MVPA during week 11 of the intervention. When each classroom met their goal, a new goal was implemented to challenge more students to increase their intensity level during activity breaks. Figure 1 displays the daily, weekly, and post-intervention incentives. Based on the teachers’ history of frequently using “student of the day” designations to reinforce positive behavior among students in the classroom, we used the same strategy for the daily reward and included an opportunity to win a personalized t-shirt. Based on previous research in the gamification literature where the use of badges prompted continued participation among study participants, we chose trophies and stickers as weekly rewards [28]. Finally, based on student and teacher feedback, the choice of a 2 h field day was strongly endorsed and used as a post-intervention reward. 

### 2.5. Instruments: Measure of the Physical Activity Intensity 

Direct observation via the System for Observing Play and Leisure Activity in Youth (SOPLAY) was used to assess PA participation and intensity level of activity breaks [29]. Over repeated time intervals, SOPLAY uses visual scans of the classroom from left to right at a rate of one person per second followed by noting characteristics of activity levels within the area. Activity levels were coded as sedentary (e.g., sitting, standing, or lying down), light activity (e.g., walking), and moderate-to-vigorous activity (e.g., more than walking). SOPLAY was designed to record PA levels in open environments and has been shown to be highly reliable in youth [29]. Separate scans were completed for boys, girls, and children with asthma. For the purposes of this analysis, only scans for boys and girls were included (*n* = 205), as separate scans by gender for children with asthma were not conducted.

A total of six trained InPACT research team members visited each classroom for approximately 4 h per visit to directly observe activity breaks. There was one observer per class except when inter-rater reliability occurred, wherein two observers were present in the class. The inter-rater reliability was 0.76 (boys) and 0.88 (girls) across all observational scans. A total of 294 observations were recorded from all classrooms combined. 

### 2.6. Data Analyses

During each week of the standard and gamified interventions, the percent of students participating in MVPA was calculated by dividing the number of students participating in MVPA during an activity break by the total number of students in the classroom. These calculations were completed for all classrooms combined, by grade level and gender. 

### 2.7. Statistical Analyses

A generalized linear mixed model with a negative binomial regression was used to assess changes overtime in the percent of students participating at the desired moderate-to-vigorous intensity level during the gamified activity breaks. Fixed effects were week, intervention (standard vs. gamified), grade level (third vs. fourth vs. fifth vs. sixth), week × intervention, and week × grade level. Random factors were the intercepts. The number of students in the classrooms was included in the analysis. Analyses were conducted for all classrooms combined, by grade level and gender, with standard intervention, sixth grade, and boys used as the reference groups. 

To depict the percentage of students in MVPA at the end of the standard and gamified interventions, a paired *t*-test was used to assess significant differences in MVPA participation for all classrooms combined, by grade level and gender. All analyses were conducted using STATA 15.0 (Statacorp, Lakeway, TX, USA) and significance was set at *p* < 0.05. 

## 3. Results

### Physical Activity Intensity 

**Figure 2:**
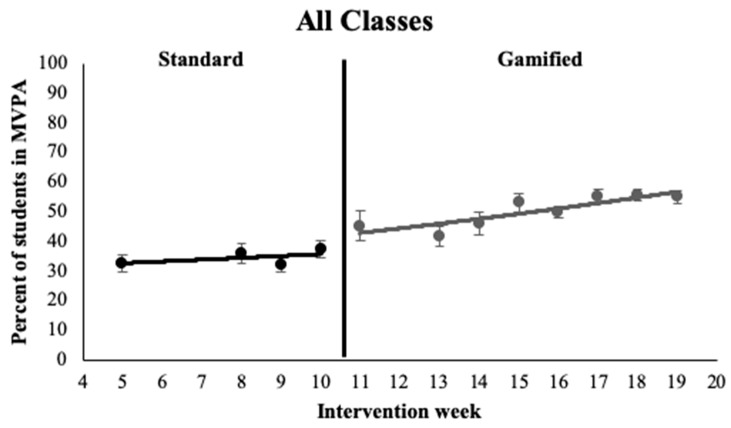
Exponential trendlines for the average student moderate-to-vigorous physical activity (MVPA) participation during an activity break, during the standard and gamified weeks of the intervention, for all classes combined.

Figure 2 displays the average percentage of students participating in MVPA during an activity break during the standard and gamified intervention weeks. For all classrooms combined, at the start of the standard intervention, 25.3% ± 0.20% of students were engaging in MVPA during an activity break. There was a significant effect of intervention, with a 27.3% ± 0.11% increase in student engagement in MVPA during an activity break during the gamified intervention weeks compared with the standard intervention weeks (*p* = 0.03). During the gamified intervention, there was a 4.9% ± 0.04% increase in MVPA participation per week, however, this change was insignificant (*p* > 0.05).

For third- and fifth-grade classrooms, significantly more students were engaged in MVPA during activity breaks compared with sixth-grade students at the start of the standard intervention, (third grade: 41.6% ± 0.21%; fifth grade: 44.4% ± 0.21%; sixth grade: 15.4% ± 0.21%; *p* = 0.01). Fourth-grade classrooms did not significantly differ from sixth-grade classrooms (fourth grade: 16.43% ± 0.22%; sixth grade: 15.4% ± 0.21%; *p* > 0.05). During the gamified intervention, the weekly increase in student MVPA participation was significantly smaller for the third- and fifth-grade classrooms compared with sixth-grade classrooms (third grade: 0.5% ± 0.04%; fifth grade: 1.0% ± 0.04%, sixth grade: 9.0% ± 0.04%; *p* = 0.02). There were no significant differences in the weekly increase in MVPA participation between fourth- and sixth-grade classrooms during the gamified intervention (fourth grade: 8.7% ± 0.04%; sixth grade: 9.0% ± 0.04%; *p* > 0.05). 

For girls and boys, there were no significant differences in student participation in MVPA during an activity break at the start of the standard intervention (girls: 30.3% ± 0.12%; boys: 29.5% ± 0.12%; *p* > 0.05). However, there was a larger increase in student MVPA participation per week throughout the gamified intervention weeks for girls compared with boys, with girls increasing MVPA participation by 5.4% ± 0.03% each week and boys increasing MVPA participation by 2.1% ± 0.03% each week (*p* > 0.05). 

Table 1 depicts the percentage of students in MVPA during the last week of the standard intervention and during the last week of the gamified intervention. There was a significant increase in student MVPA participation during an activity break between the last week of the standard and gamified interventions (*p* = 0.01). There was also a significant increase in student MVPA participation during an activity break in fourth through sixth grade between the last week of the standard and gamified interventions (every *p* < 0.05); however, there were no significant changes among third-grade students (*p* > 0.05). In addition, there was a significant increase in girls and boys MVPA participation during an activity break between the last week of the standard and gamified interventions (standard: 43.4% ± 4.7%, gamified: 57.0% ± 3.1%, *p* = 0.01; boys standard: 31.4% ± 3.4%, gamified: 53.2% ± 3.0%, *p* = 0.01). 

Six out of nine classrooms met their MVPA weekly goal 6 out of the 7 possible weeks and, therefore, were awarded the prize of attending the InPACT field day (2 h of structured PA outdoors). As for the three classrooms that did not earn the final prize, one third-grade class met their goal 4 out of 7 weeks, one fourth-grade class met their goal 5 out of 7 weeks, and one sixth-grade class met their goal 4 out of 7 weeks. 

## 4. Discussion

The present study determined the effect of adding game design elements guided by goal-setting theory to an evidence-based intervention, InPACT, to enhance in-class PA participation among elementary school age students. Specifically, goal-setting theory was used to gamify InPACT by setting attainable classroom goals, providing weekly feedback on student progress toward meeting the goal, and establishing an end-of-study reward to motivate behavior change towards increased PA levels. Our findings demonstrated that gamification increased the percentage of students exercising at a moderate-to-vigorous intensity during an activity break by 27%. Additionally, gamification increased student MVPA participation in all grades and in both boys and girls, with greater increases observed over time in fourth- and sixth-grade classrooms and female students. These findings suggest gamification is a tool that can be integrated into classroom-based PA interventions to enable children to accumulate health-enhancing PA in this environment.

Gamification has previously been applied to pediatric PA interventions outside of the classroom. For example, Verstraete et al. provided game equipment during recess and demonstrated a significant increase in MVPA (from 48% to 61%) in the intervention group, while activity levels decreased in the control group [30]. In the Beat the Street intervention, Coombes et al. examined the use of gamification on active transport to school in children aged 8–10 years by providing rewards such as supplying the school with books [31]. Coombes et al. demonstrated that total MVPA increased during active transport by approximately 3.5 min in children who engaged in the intervention at least 15 out of 63 days [31]. While these authors noted a significant increase in MVPA participation through the provision of rewards, it was unclear how their incentive structures were informed by theory. The present study was guided by goal-setting theory and designed to include setting specific and achievable goals as well as receiving weekly feedback and rewards. This theory-based design resulted in a 27% increase in student MVPA participation during the gamified activity breaks compared with standard activity breaks. At the end of the intervention, approximately 55% of students were exercising at the desired intensity, thereby accumulating 20 min of health-enhancing PA per day in the classroom. This 27% increase in student MVPA was similar to that found by Althoff et al., who demonstrated a 26% increase in daily step counts following the use of the Pokémon Go™ app over a period of 30 days [16]. Future research should seek to determine the independent effect of each game design element (goal setting, feedback, and rewards) in motivating behavior change toward increased PA participation at the desired intensity among children. 

While gamification increased MVPA participation during activity breaks across all grades, a smaller improvement was observed in the third- and fifth-grade classrooms. A key tenet of the goal-setting theory is that individuals must accept the goals they are given [12]. Given the higher baseline participation in these classrooms, students may have benefited from a more challenging goal to motivate an increase in MVPA participation. Future studies should consider including student feedback when developing goals. Another possible reason for the smaller improvements noted in third- and fifth-grade classrooms may relate to the type of rewards provided. Cugelman et al. suggests that the type of rewards offered during an intervention is an important motivating factor for behavior change because the reward may or may not be of value to the community or individual participants [32]. Specifically, Cugelman et al. suggest it is important to identify and tailor specific intervention components that will be receptive to the population and setting. As such, the rewards offered during InPACT (i.e., placing a series of stickers on their class trophy in order to win a field day) may not have appealed as motivational factors to students in third- and fifth-grade classrooms. In addition, other factors that may have contributed to a lack of motivation in these classrooms include: potential differences in social connectedness, perceptions of fun, and capacity to overcome challenges, all of which can affect student motivation [32]. Additional research is needed to better understand the grade-level differences in student motivation for MVPA related to goal setting and external rewards given during gamified activity breaks. 

Previous classroom-based PA interventions have shown boys and girls participate at similar rates [4,8,9]. For example, Bartholomew et al. found that gender did not moderate the effect of step count and MVPA participation during the Texas-Initiatives for Children’s Activity and Nutrition (Texas-ICAN) intervention [8]. Similarly, during the first iteration of InPACT, boys and girls participated in MVPA during activity breaks at similar rates [9]. In the present study, gamification appeared to be a motivator for both boys and girls, with boys and girls increasing their participation in MVPA throughout the gamified intervention. Providing specific, attainable, yet challenging goals may be a strategy to motivate both boys and, importantly, girls participation levels during classroom activity breaks [33]. While both boys and girls increased MVPA participation, we observed a slightly larger increase over the gamified weeks in girl’s participation compared to that of boys. The mode of exercise (92% of activity breaks implemented were dance videos) may in part account for larger increase in participation observed in girls. Schmalz and Kerstetter examined participation rates of boys and girls in dance and found only 2% of boys in the sample, aged 8–10 years, participated in dance compared with 62% of girls [34]. It is also important to note that classroom activity breaks address many of the gender-based barriers to PA experienced by girls in other school-based PA programs. In a focus group of 34 African-American and Latina elementary-age children, Taylor et al. examined perceived barriers for PA participation in girls. Two of these barriers were (1) appearance (e.g., not wanting to get sweaty) and (2) lack of opportunities (e.g., boys not sharing equipment) [35]. Classroom activity breaks address these barriers, as they do not require equipment and are short in duration (i.e., 4 min), meaning a smaller chance of sweating. As such, gamified classroom activity breaks may help to reduce gender disparities in PA participation while also increasing opportunities for both boys and girls to be physically active at school. 

A few strengths of this study should be noted. First, gamification protocols were grounded in goal-setting theory and applied to a classroom-based PA intervention for the first time. Second, the use of direct observation for measuring activity intensity is more robust than methods such as survey self-responses. The present study also had limitations. First, the absence of control classrooms did not allow for direct comparison of gamified versus non-gamified activity breaks in this sample population. Second, the increase in student participation may have been the result of the novelty of the intervention broadly rather than the specific gamified qualities. This is unlikely, however, since the percentage increase in MVPA participation during activity breaks was maintained throughout the gamification period. Third, we acknowledge the sample of classrooms was not randomly selected and, therefore, teachers may have had biased views of PA. Fourth, students were aware of the days when the research team would be present to observe, potentially influencing participation rates. Fifth, we were primarily focused on providing challenging and attainable weekly goals that empowered students to achieve their class goal rather than focused on obtaining a statistically significant increase in MVPA participation. Therefore, the 5%–10% weekly increase may not have been a large enough goal to observe a statistically significant change over time. Lastly, we did not collect a measure of PA participation outside of the classroom (e.g., recess, physical education, afterschool sports, and weather) and cannot determine the potential influence of this factor on our intervention findings. Future research should incorporate accelerometer data to assess total daily PA behavior to better understand the independent intervention effects on MVPA participation among students.

## 5. Conclusions

Applying gamification protocols to classroom-based PA interventions is an important and promising strategy to consider when promoting PA participation during the school day. This study infused game design elements guided by goal-setting theory (i.e., goal setting, feedback, and external rewards) into a PA intervention, resulting in 55% of students accumulating approximately 20 min of health-enhancing PA in the classroom per day. It is important to note that an additional 30% of students were participating in the activity breaks but at a lower intensity, with only 15% of children not regularly participating. Hence, additional strategies are needed to maximize participation in classroom activity breaks in under-resourced schools. Nevertheless, gamification may be a key tool to decrease the gap in participation previously observed among students in schools of varying socioeconomic status. Future research should continue to investigate the effects of gamification on PA participation as well as examine the independent effects of each game design element to motivate behavior change and determine whether gamification results in a sustained change in PA participation over time. 

## Figures and Tables

**Table 1:** Average student moderate-to-vigorous physical activity participation outcomes for all classes combined, by grade level and gender, for the last week of the standard and gamified intervention.

Percent of Students in Moderate-to-Vigorous Physical Activity (MVPA) during an Activity Break				
	Grade Level	Standard	Gamified	*p*-Value
		(Mean ± SE)	(Mean ± SE)	
	3	53.3 ± 6.5	64.3 ± 3.9	0.13
	4	12.5 ± 2.6	48.4 ± 3.0	0.01
	5	46.0 ± 3.8	62.9 ± 6.2	0.02
	6	26.1 ± 6.7	47.6 ± 5.3	0.05
**Overall**	--	37.3 ± 3.0	55.1 ± 2.1	0.01
**Boys**	--	31.4 ± 3.4	53.2 ± 3.0	0.01
**Girls**	--	43.4 ± 4.7	57.0 ± 3.1	0.01
